# Admission haemoglobin and albumin as correlates of central nervous system inflammation in *rhodesiense human African trypanosomiasis* at Rumphi District Hospital, Malawi

**DOI:** 10.4102/jphia.v17i1.1775

**Published:** 2026-08-28

**Authors:** Westain T. Nyirenda, Lovemore Mapahla, Bonheur Dounebaine, Nebiyu Dereje, Peter S. Nyasulu

**Affiliations:** 1 Division of Epidemiology and Biostatistics, Department of Global Health, Faculty of Medicine and Health Sciences, Stellenbosch University, Cape Town, South Africa; 2 Africa Centres for Disease Control and Prevention, Addis Ababa, Ethiopia; 3 School of Public Health, Wachemo University, Hosainna, Ethiopia; 4 Division of Epidemiology and Biostatistics, Faculty of Medicine and Health Sciences, University of the Witwatersrand, Johannesburg, South Africa

**Keywords:** *rhodesiense human African trypanosomiasis*, sleeping sickness, central nervous system involvement, haemoglobin, Albumin, cerebrospinal fluid white-cell count

## Abstract

**Background:**

*Rhodesiense human African trypanosomiasis* (*rHAT*) is an acute zoonotic infection that can rapidly progress to central nervous system (CNS) disease. As lumbar puncture is no longer routinely required for treatment selection, district hospitals need accessible measures associated with CNS inflammatory burden.

**Aim:**

This study aimed to evaluate routine admission clinical and laboratory measures as correlates of cerebrospinal-fluid white-cell count and to describe changes in this count during early hospital care.

**Setting:**

The study was conducted at Rumphi District Hospital, a public secondary-level treatment facility serving rural communities in the Vwaza Marsh focus of northern Malawi.

**Methods:**

We retrospectively studied patients aged ≥ 6 years with parasitological confirmed *rHAT* from 2012–2024. Cerebrospinal-fluid white-cell counts at admission and Day 11 were analysed using mixed-effects negative binomial regression, reporting adjusted incidence rate ratios (aIRRs) with 95% confidence intervals (CIs).

**Results:**

Forty-three patients met the eligibility criteria; 43 contributed admission counts, and 42 contributed Day-11 counts. Day-11 cerebrospinal-fluid white-cell counts were 75% lower than admission counts (aIRR = 0.25, 95% CI: 0.14–0.45; *p* < 0.001). Stage 2 disease was associated with a higher count than Stage 1 disease (aIRR = 4.23, 95% CI: 1.35–13.30; *p* = 0.013). Each 1 g/dL increase in haemoglobin and albumin was associated with lower expected counts (haemoglobin aIRR = 0.83, 95% CI: 0.78–0.89; *p* < 0.001; albumin aIRR = 0.70, 95% CI: 0.54–0.92; *p* = 0. 009).

**Conclusion:**

Lower haemoglobin and albumin were associated with greater cerebrospinal-fluid inflammation, which declined during early care. As accessible admission measures, they may support recognition, triage and monitoring of CNS burden in *rHAT*, pending prospective diagnostic-accuracy validation before implementation.

**Contribution:**

This study links routine blood measures to cerebrospinal-fluid inflammation in *rHAT*, informing prospective validation priorities.

## Introduction

*Human African trypanosomiasis (HAT)* remains a neglected tropical disease that imposes substantial individual and health-system burdens despite major declines in incidence over the past two decades, driven by strengthened surveillance, vector control and improved case management.^1,2,3^ Two subspecies of *Trypanosoma brucei* cause HAT (Tb HAT): *T. b. gambiense* in West and Central Africa (typically chronic) and *T.b. rhodesiense human African trypanosomiasis* (*rHAT*) in East and Southern Africa (typically acute and zoonotic).^1,4,5,6^ Although *rHAT* now constitutes a smaller share of global cases, it progresses rapidly and is frequently severe at presentation; delayed treatment is associated with high mortality and neurological sequelae. Endemic districts in Malawi, Tanzania, Uganda and Zambia continue to report cases, and *rHAT* features prominently among imported infections in travellers and expatriates.^5,6^

Historically, clinical staging anchored programme workflows: haemolymphatic (Stage 1) versus meningo-encephalitic (Stage 2) disease, classified by detection of trypanosomes in cerebrospinal fluid (CSF) and/or elevated CSF white-cell count (CSF WCC).^6,7,8^ Yet CSF staging is operationally demanding in peripheral hospitals, requiring trained staff, sterile consumables, microscopy and patient consent. Moreover, fixed CSF WCC thresholds of ≥ 5 cells/μL only imperfectly reflect the biology and spectrum of central nervous system (CNS) involvement and vary across programmes.^5,6,9,10^ Recent World Health Organization guidance has expanded the use of oral fexinidazole for *rHAT* and has reduced reliance on routine lumbar puncture for initial treatment decisions. In this context, rapid and objective triage signals remain essential in peripheral facilities.^11,12^ In this evolving therapeutic era, clinicians still need pragmatic, field-ready indicators to recognise patients with substantial CNS inflammatory burden, prioritise monitoring, referral and anticipate complications when CSF analysis is not prioritised.

The clinical phenotype of *rHAT* at presentation is well described-fever, headache, lymphadenopathy, hepatosplenomegaly and early neuropsychiatric features-with reports from Tanzania and Uganda highlighting the rapid tempo towards Stage 2 disease.^7,10^ However, fewer studies have quantified how simple admission measurements track with objective CNS inflammation. Routine markers such as haemoglobin, leukocyte and platelet counts, creatinine and serum albumin are available in many district hospitals and may provide actionable signals. Biologically, anaemia of inflammation (hepcidin-mediated iron sequestration and marrow suppression) and hypoalbuminaemia (negative acute-phase response and capillary leak) plausibly accompany severe systemic and intrathecal inflammation.^13,14,15^ Metabolomic and proteomic studies in *HAT* further support systemic inflammatory and metabolic perturbations during CNS involvement, but these platforms are not yet programmatically scalable, and district-level evidence leveraging routine tests remains sparse and sometimes inconsistent.^6,11,12^

Against this backdrop, we examined whether routinely collected admission measures can serve as correlates of CNS inflammatory burden in *rHAT*. Using hospital data from Rumphi District Hospital (RDH) (northern Malawi), we tested the association between CSF WCC (a pragmatic proxy for CNS inflammation) and candidate clinical/laboratory predictors, with particular interest in haemoglobin and serum albumin as feasible triage markers. We also quantified short-term in-hospital trajectories in CSF WCC between admission (Day 0) and Day 11. Our research question was: *Among patients with parasitologically confirmed rHAT, which readily available admission measures are independently associated with CSF WCC and stage at presentation, and how does CSF WCC evolve over the initial hospital course?* We targeted CNS inflammatory burden directly by modelling CSF WCC over time. This is because Stage 2 incorporates a CSF WCC threshold, stage was treated as a derived proxy used for adjustment/validation rather than as a separate outcome to avoid circularity. By focusing on scalable markers in a district-hospital setting, this study aims to inform triage pathways and bedside risk stratification in resource-limited programmes where routine lumbar puncture is not emphasised.^11,12^

## Research methods and design

### Study design

We conducted a retrospective cohort study using hospital data obtained from patients admitted to RDH, in the northern region of Malawi between January 2012 and December 2024. The patients were followed up on Day 5 and Day 11 of hospitalisation.

### Study setting

Rumphi District Hospital is a public, secondary-level facility in northern Malawi serving a predominantly rural population. The hospital provides inpatient and outpatient care, routine haematology and biochemistry, and microscopy for parasitological confirmation of *rHAT*. Rumphi District Hospital serves a rural catchment bordering Vwaza Marsh Wildlife Reserve, a recognised focus of *T. brucei rHAT* in northern Malawi; WHO Global Health Observatory data provide the national reporting framework for annual *rHAT* case counts.^1,4^ An evaluation conducted from August 2014 to July 2017 identified 78 cases in the focus, including six deaths, and showed that bringing diagnostic services closer to at-risk communities promoted earlier diagnosis and improved outcomes.^16^ A subsequent One Health analysis estimated an incidence of 5.9 hospital-diagnosed human trypanosomiasis cases per 100 000 people in Rumphi District.^17^ These data support the continued relevance of RDH as a sentinel treatment and surveillance site.

### Study population and sampling strategy

We included all patients aged ≥ 6 years admitted to RDH (northern Malawi) with parasitologically confirmed *T. brucei rHAT* between 01 January 2012 and 31 December 2024, using national and WHO diagnostic criteria: trypanosomes detected by microscopy in blood or CSF. All the included patients received the same treatment, Fexinidazole. We excluded records with incomplete data defined as missing CSF WCC and predetermined covariate results at admission, Day 5 and Day 11 time-points.

This was a retrospective cohort study of all eligible admissions from 01 January 2012 to 31 December 2024. Therefore, no a priori sample-size calculation was performed. Furthermore, in line with current recommendations, we do not present post hoc power,^18^ because post hoc power estimates are mathematically determined by the observed effect size and p-value. They add no information beyond the reported confidence intervals (CIs), for completeness, we therefore report precision via 95% CI rather than post hoc power.^18^

### Data collection

Data were obtained from patient files, ward registers and laboratory logs using a standardised Excel spreadsheet. Clinical and laboratory data were recorded at up to two analytic time points: Baseline (Day 0) and Day 11 (end of hospitalisation). Day 5 values were variably documented and were not used for the primary outcome.

We defined all variables prior to data capturing. Age (years) was calculated at admission from the date of birth or recorded stated age; the admitting clinician recorded sex (female/male). Symptoms and examination findings were transcribed as documented (present/absent) at admission. For synthesis, we defined neurological symptoms and signs as the presence of ‘drowsiness, reduced level of awareness (lethargy), or difficulty walking; and lymphatic involvement as the presence of cervical adenomegaly and/or splenomegaly on examination’. Vital signs reflected the first set obtained at admission: heart rate (beats per minute) by pulse/monitor, systolic/diastolic blood pressure (mmHg) by automated or manual sphygmomanometer, respiratory rate (breaths per minute) by direct count, and temperature (°C) by digital thermometer per ward protocol. Functional status used the clinician-assigned Karnofsky Performance Status (0–100, higher scores indicate better function) and Glasgow Coma Scale.^3,4,5,6,7,8,9,10,11,12,13,14,15^ Routine laboratory tests were measured on same-day venous blood using the hospital’s automated analysers according to laboratory standard operating procedures: haemoglobin (g/dL), total leukocyte count (×10^9^/L), platelet count (×10^9^/L), serum creatinine (mg/dL) and serum albumin (g/dL). The primary outcome, CSF WCC, was reported as cells/μL from laboratory registers following the laboratory’s microscopy-based cell counting procedure; repeat CSF counts at Day 11 were extracted from the records. When multiple measurements were available on the same day, the value closest to the admission time was used; time-varying covariates such as haemoglobin and albumin used the concurrent value at each modelled time point. Binary variables were coded with explicit reference categories for analysis as specified in the statistical analysis section. Disease staging of *rHAT* followed trypanosomiasis case management guidelines (Stage 2: trypanosomes in CSF and/or CSF WCC > 5 cells/μL).^19^ Data were entered into a structured spreadsheet with a prespecified codebook, harmonised units and labelled categorical values (e.g., sex: female/male; Stage: 1/2). Source documents were linked by a unique study ID; direct identifiers were removed before analysis. Range and logic checks flagged implausible values for verification against source documents. Data sets and documentation were stored on a secure, password access-restricted drive.

### Data analysis

Baseline characteristics were summarised overall. Distributional form was assessed using the Shapiro–Wilk test and graphical inspection. Normally distributed continuous variables are reported as mean (standard deviation), non-normally distributed continuous variables as median (interquartile range [IQR]), and categorical variables as number (percentage). All tests were two-sided with α = 0.05.

This study evaluated associations between routinely measured clinical and laboratory variables and cerebrospinal-fluid white-cell count; it was not designed as a clinical prediction or diagnostic-accuracy study. Discrimination, calibration, receiver operating characteristic curves, cut-off values, positive predictive values and negative predictive values were therefore not estimated. Because Stage 2 disease is partly defined by cerebrospinal-fluid white-cell count, using stage as the reference standard for receiver operating characteristic analysis would introduce incorporation bias. The findings should consequently be interpreted as hypothesis-generating correlates that require prospective validation against an independent clinical reference standard.

The primary outcome was CSF WCC (cells/μL), analysed as a count. We fitted prespecified mixed-effects negative binomial regression models with a log link and a patient-level random intercept to account for within-patient correlation across baseline and Day 11; if the negative binomial failed to converge or showed negligible over-dispersion, we used a Poisson model with robust (sandwich) standard errors as a sensitivity check. Results are reported as incidence rate ratios (IRRs) with 95% CIs and two-sided *p*-values. We selected candidate variables by prespecifying candidate correlates on clinical and programmatic grounds: age, sex, assessment time point (Day 11 vs. Baseline), malaria test (positive vs. negative), disease Stage (Stage 2 vs. Stage 1), haemoglobin, creatinine, Karnofsky Performance Status (per point), albumin, leukocytes, respiratory rate, facial oedema (yes vs. no) and platelets. Age, sex, time point and stage were retained in all models irrespective of statistical significance. Remaining variables entered the multivariable model if their univariable mixed-effects count regression met *p* < 0.05. Time-varying covariates used values measured at each time point. Disease Stage (2 vs. 1) was entered as a fixed covariate to establish criterion validity (Stage 2 ↔ higher CSF WCC). We did not specify stage as a primary outcome because its definition incorporates CSF WCC (incorporation bias). Instead, modelling CSF WCC as a count preserved information, enabled mixed-effects inference across baseline and Day 11, and avoided loss of power from dichotomisation. Where multiple correlation screens were performed, we present both raw and Benjamini–Hochberg false discovery rate (BH-FDR)–adjusted *p*-values.^20,21^

We assessed over-dispersion and compared negative-binomial with Poisson specifications Akaike Information Criterion (AIC) and Bayesian Information Criterion (BIC), likelihood-ratio tests), when the negative-binomial alpha approached zero, or convergence warnings arose, we fitted Poisson models with robust (sandwich) standard errors as a sensitivity check. Intraclass correlation coefficients showed substantial clustering (empty model Intraclass Correlation Coefficient [ICC] ≈ 0.45) and supported random effects after adjustment (conditional ICC ≈ 0.98; random-intercept variance 3.42). Variance-inflation factors flagged collinearity for visit day and Karnofsky (Variance-inflation Factor [VIF] > 5); sensitivity analyses excluding these terms did not change inference. Residual and influence checks did not indicate mis-specification. All tests were two-sided (α = 0.05), and we report incidence rate ratios with 95% CIs and *p*-values. Except for sex and respiratory rate, completeness exceeded 98%, so we used complete-case analysis; all analyses were performed in R (v4.2.3).

### Ethical considerations

This retrospective study analysed anonymised hospital data and involved no direct contact with patients. Access to source hospital records was authorised by Rumphi District Hospital. The study protocol was reviewed and approved by the Stellenbosch University Health Research Ethics Committee (HREC; approval S24/11/324), which granted a waiver of individual informed consent in line with national regulations. All procedures complied with the Declaration of Helsinki and applicable local governance requirements.

## Results

### Patient demographics and clinical characteristics

**FIGURE 1:**
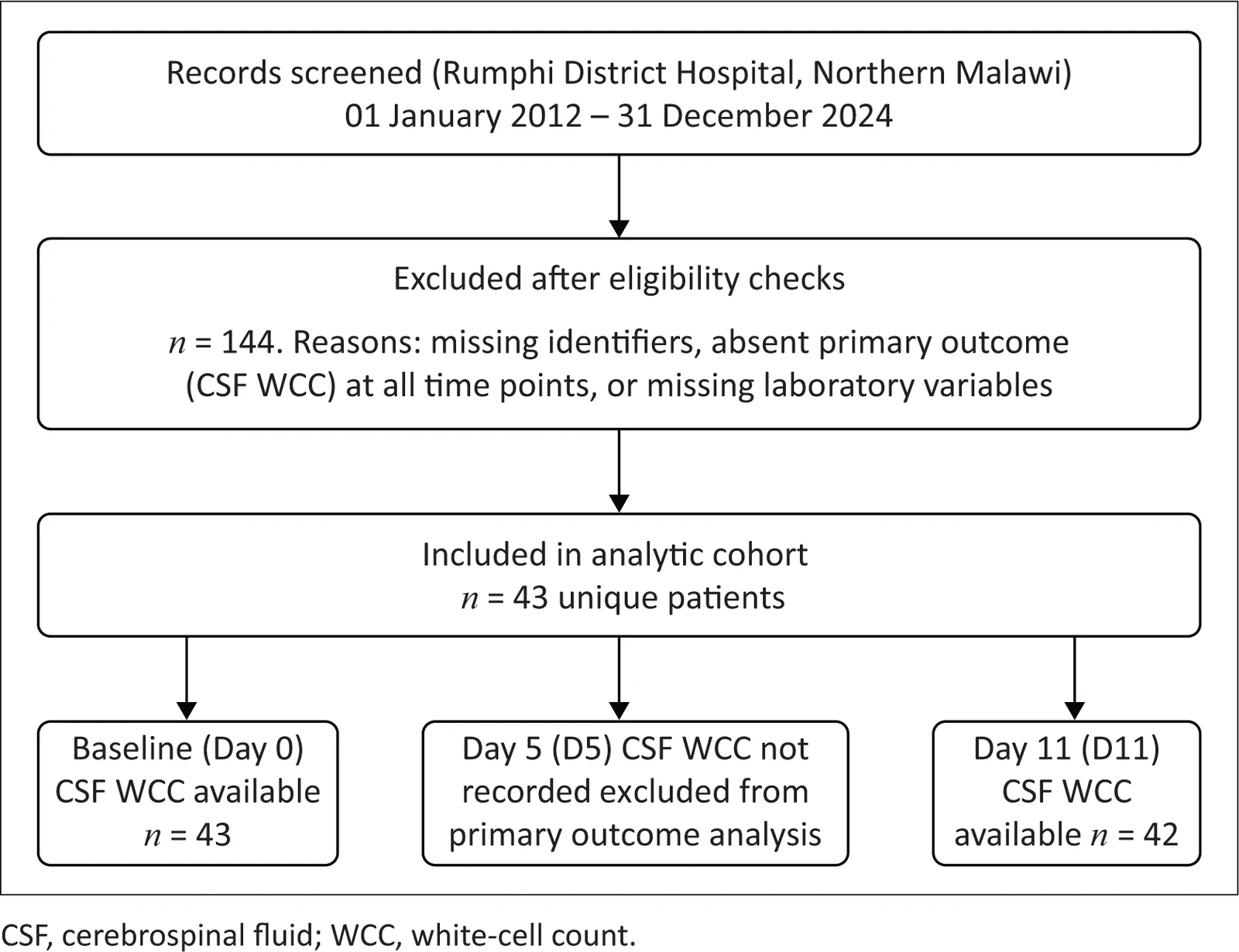
Study cohort profile and data availability for *rhodesiense human African trypanosomiasis*: Rumphi District Hospital, Northern Malawi (2012–2024).

A total of 43 patients aged at least 6 years with parasitologically confirmed *rHAT* met the eligibility criteria (Figure 1). All 43 contributed admission data and 42 contributed Day-11 cerebrospinal-fluid white-cell counts. At admission, the median cerebrospinal-fluid white-cell count was 8 cells/μL (IQR: 3–59.5; range: 0–363).

### Clinical characteristics during *rhodesiense human African trypanosomiasis* patient admission

Out of the 43 patients included in the analysis, 32.6% (*n* = 14) were female and 67.4% (*n* = 29) were male. The median age at presentation was 25 years (IQR: 17.5–40.5). All patients had confirmed *T. brucei* rhodesiense infection with baseline clinical and laboratory data available. All participants were residents of Vwaza catchment area. Most patients were admitted through the medical ward, which is consistent with routine case management pathways for *rHAT* at district level.

The categorical demographics and clinical characteristics are summarised in Table 1. Fever and headache were recorded in 42 (98%) and 41 (95%) patients, respectively. The composite neurological variable – drowsiness, impaired awareness or walking difficulty – was present in 39 (91%). Malaria co-infection was documented in 10 (23%), facial oedema in 12 (28%) and lymphatic involvement in 19 (44%). Because each symptom was assessed independently, percentages across symptoms are not mutually exclusive and should not be summed.

**TABLE 1:** Baseline categorical demographic and clinical characteristics of patients with *rhodesiense human African trypanosomiasis* admitted to Rumphi District Hospital, Malawi (2012–2024).

Characteristic	*n* †	%
Sex: Female	14	33
Sex: Male	29	67
Headache	41	95
Neuro symptoms and signs‡	39	91
Fever	42	98
Insomnia	25	58
Pruritus	13	30
Diarrhoea	2	4.7
Malaria	10	23
Weight loss	12	28
Facial oedema	12	28
Lymphatic involvement§	19	44

Note: Symptom frequencies were assessed independently across the cohort (*N* = 43 for each symptom).

† , *N* = 43 is the denominator for each variable;

‡ , Neuro symptoms and signs: drowsiness and or difficult walking and or lethargy;

§ , Lymphatic involvement: cervical adenomegaly and or splenomegaly at baseline.

On physical examination, 28% had facial oedema and 44% had lymphoreticular signs, which included splenomegaly and cervical adenomegaly. Laboratory assessments at baseline showed anaemia (median haemoglobin 9.6 g/dL, IQR: 7.5–11.3) and hypoalbuminaemia (median albumin 2.38 g/dL, IQR: 2.1–2.6), consistent with systemic illnesses such as human immunodeficiency virus (HIV) and/or acquired immunodeficiency syndrome (AIDS) and tuberculosis (TB). Malaria co-infection was the most frequent comorbidity (23%).

All 43 patients received fexinidazole as first-line treatment. By discharge, 42 out of 43 patients (97.7%) had completed treatment, while 1 out of 43 (2.3%) died from complications judged unrelated to fexinidazole. One patient (2.3%) developed treatment non-response and received melarsoprol as second-line therapy.

The continuous baseline characteristics are presented according to their distributions. Variables summarised as mean (standard deviation) are shown in Table 2, while variables summarised as median (IQR), including cerebrospinal-fluid white-cell count, are shown in Table 3.

**TABLE 2:** Baseline continuous demographic, physiological, functional and laboratory characteristics of patients with *rhodesiense human African trypanosomiasis* admitted to Rumphi District Hospital, Malawi (2012–2024).

Characteristic	Mean ± s.d.	Median	IQR
Age (years)	-	25.0	17.5–40.5
Systolic BP (mmHg)	102.23 ± 17.08	-	-
Diastolic BP (mmHg)	61.07 ± 11.57	-	-
Heart rate (bpm)	106.09 ± 16.67	-	-
Respiratory rate (/min)	-	23.0	20.0–24.0
Temperature (°C)	37.46 ± 1.13	-	-
BMI (kg/m^2^)	18.90 ± 3.76	-	-
Karnofsky index	-	70.0	70.0–80.0
GCS	-	15.0	15.0–15.0
Haemoglobin (g/dL)	9.60 ± 2.74	-	-
Platelets (×10^9^/L)	130.56 ± 81.64	-	-
Leukocytes (×10^9^/L)	-	4.50	3.35–5.45
Albumin (g/dL)	2.38 ± 0.47	-	-
Creatinine (mg/dL)	-	0.80	0.50–1.00
Glucose (mg/dL)	-	102.50	86.25–116.00
CSF WCC (cells/mL)	-	8	3–59.5

Note: Data are median (IQR); or mean (s.d.), as indicated. *N* = 43 is the denominator for each variable.

IQR, interquartile range; BMI, body mass index; CSF, cerebrospinal fluid; WCC, white-cell count; s.d., standard deviation; BP, blood pressure; GCS, Glasgow Coma Scale.

**TABLE 3:** Univariable mixed-effects negative binomial regression of factors associated with cerebrospinal fluid white-cell count among hospitalised patients with *human African trypanosomiasis* at Rumphi District Hospital, Malawi (2012–2024).

Characteristic	IRR	95% CI	*p*-value
Age (per year)	0.97	0.94–1.00	0.031
Sex			
Female (ref)	-	-	-
Male	0.78	0.27–2.24	0.600
Visit day			
Day 0 (ref)	-	-	-
Day 11	0.34	0.31–0.36	< 0.001
Platelets (×10^9^/L)	1.00	1.00–1.00	< 0.001
Malaria test			
Negative (ref)	-	-	-
Positive	3.59	1.19–10.8	0.023
*rHAT* stage			
1 (ref)	-	-	-
2	8.37	2.97–23.6	< 0.001
Haemoglobin (g/dL)	0.63	0.60–0.65	< 0.001
Creatinine (mg/dL)	0.71	0.60–0.85	< 0.001
Karnofsky index (per 1-point)	0.97	0.97–0.97	< 0.001
Albumin (g/dL)	0.34	0.31–0.39	< 0.001
Leukocytes (×10^9^/L)	0.62	0.60–0.65	< 0.001
Respiratory rate (per min)	0.98	0.96–1.00	0.093
Facial oedema			
No (ref)	-	-	-
Yes	0.72	0.60–0.88	0.001

Note: Estimates from univariable mixed-effects count models (negative binomial preferred; robust Poisson when needed), with a patient-level random intercept to account for repeated measures. Continuous predictors are per-unit increase as shown. Reference (ref) categories are indicated.

IRR, incidence rate ratio; CI, confidence interval; ref, reference; *rHAT, rhodesiense human African trypanosomiasis*.

### Associations of high CSF-WCC at presentation and over hospitalisation

In univariable mixed-effects negative binomial models, cerebrospinal-fluid white-cell count was statistically associated with age, assessment time point, platelet count, malaria test result, disease stage, haemoglobin, creatinine, Karnofsky Performance Status, albumin, peripheral leukocyte count and facial oedema (all *p* < 0.05). There was no evidence of association at the 5% level for sex or respiratory rate. Complete effect estimates and CIs are presented in Table 4. Because these estimates are unadjusted, interpretation is based primarily on the multivariable model.

**TABLE 4:** Multivariable mixed-effects negative binomial regression of factors associated with cerebrospinal fluid white-cell count among patients with *rhodesiense human African trypanosomiasis* at Rumphi District Hospital, Malawi (2012–2024).

Characteristic	aIRR	95% CI	*p*-value
Age (per year)	0.98	0.95–1.01	0.3
Sex			
Female (ref)	-	-	-
Male	1.01	0.39–2.66	0.9
Assessment time point			
Day 0 (ref)	-	-	-
Day 11	0.25	0.14–0.45	< 0.001
Platelets (×10^9^/L)	1.00	1.00–1.00	0.058
Malaria			
Negative (ref)	-	-	-
Positive	1.96	0.67–5.71	0.2
Disease stage			
1 (ref)	-	-	-
2	4.23	1.35–13.3	0.013
Haemoglobin (g/dL)	0.83	0.78–0.89	< 0.001
Creatinine (mg/dL)	0.91	0.67–1.22	0.5
Karnofsky index (per 1-point)	1.02	1.00–1.03	0.006
Albumin (g/dL)	0.70	0.54–0.92	0.009
Leukocytes (×10^9^/L)	1.04	0.95–1.14	0.4
Facial oedema			
No (ref)	-	-	-
Yes	0.32	0.24–0.43	< 0.001

Note: *P*-values from Wald tests. Time-varying covariates (haemoglobin, albumin) use values at each time point; Rumphi District Hospital (RDH).

aIRR, adjusted incidence rate ratio; CI, confidence interval.

In the adjusted mixed-effects negative binomial model, the expected cerebrospinal-fluid white-cell count at Day 11 was 75% lower than at admission (adjusted incidence rate ratio [aIRR] = 0.25, 95% CI: 0.14–0.45; *p* < 0.001). Stage 2 disease was associated with a higher expected count than Stage 1 disease (aIRR = 4.23, 95% CI: 1.35–13.30; *p* = 0.013). Each 1 g/dL increase in haemoglobin and albumin was associated with 17% and 30% lower expected counts, respectively (haemoglobin aIRR = 0.83, 95% CI: 0.78–0.89; *p* < 0.001; albumin aIRR = 0.70, 95% CI 0.54–0.92; *p* = 0.009). Karnofsky Performance Status and facial oedema were also statistically associated with the outcome; these findings require cautious interpretation because of collinearity and possible residual confounding. Age, sex, malaria test result, creatinine and peripheral leukocyte count were not independently associated at the 5% level.

## Discussion

This study found that haemoglobin and serum albumin measured during hospital care were independently associated with cerebrospinal-fluid white-cell count in patients with *rHAT*. The decline in count by Day 11 is consistent with attenuation of intrathecal inflammation during early treatment, while the association with Stage 2 disease supports the clinical relevance of the count outcome. These observations establish association rather than prediction.

The inverse relationships of haemoglobin and albumin with cerebrospinal-fluid white-cell count are biologically plausible. Anaemia in infection is commonly driven by hepcidin-mediated iron sequestration and suppressed erythropoiesis-so-called ‘anaemia of inflammation’. As inflammatory tone rises, haemoglobin falls, and our data show that lower haemoglobin aligns with higher CSF pleocytosis, consistent with a more intense host response. Anaemia in *trypanosomiasis* can arise through inflammation-mediated iron sequestration, reduced erythropoiesis and haemolysis, whereas hypoalbuminaemia can reflect the negative acute-phase response, altered vascular permeability and nutritional compromise.^14,15,22^ Thus, patients with more pronounced systemic inflammatory disturbance may also have greater intrathecal cellular inflammation. However, the present analysis cannot determine whether changes in haemoglobin or albumin precede, cause or merely accompany changes in cerebrospinal-fluid inflammation.

Serum albumin, conversely, is a negative acute-phase reactant: systemic inflammation down-regulates hepatic albumin synthesis and shifts capillary permeability, lowering circulating albumin. Thus, hypoalbuminemia can signal systemic inflammatory and vascular-leak phenotypes that often accompany neuro-invasion in *rHAT* – again coherent with the positive association we observed between low albumin and higher CSF WCC.^23,24^

Neuroinflammation is a hallmark of late *rHAT*, with parasite traversal of the blood–brain interface and resultant CNS leukocyte recruitment. Cerebrospinal fluid pleocytosis is an accessible correlate of this biology, even as the precise mechanisms of neuro-invasion continue to be refined. The inverse relationships we found between CSF WCC and both haemoglobin and albumin fit this pathophysiological frame: patients with greater systemic inflammatory activity (anaemia of inflammation, negative acute-phase albumin) are the same patients in whom CNS inflammation is most evident.^25,26^

This study suggests that the inverse associations of haemoglobin and albumin with CSF WCC likely reflect parallel resolution of systemic and intrathecal inflammation: reduced marrow suppression and haemodilution, and less capillary leak, track with lower CSF cellularity. While our models cannot establish causality, the consistent time effect (Day 11 decline) and the associated relationships are consistent with these measures functioning as trajectory correlates in settings where lumbar puncture is not emphasised.

In practice, a patient arriving with low haemoglobin and low albumin is likely to have a higher CSF pleocytosis at that same timepoint. The observed fall in CSF WCC by Day 11 accords with expected early treatment response; clinically, one would anticipate a gradual ‘normalisation’ of systemic correlates (rising haemoglobin/albumin) as meningeal inflammation abates. These dynamics suggest that, taken together, haemoglobin and albumin could be incorporated into future triage frameworks as supportive severity-associated markers, while prospective studies define clinically actionable thresholds and confirm their utility in routine care.

The strong positive association between Stage-2 classification and CSF WCC was expected and serves as an internal validity check. The reduction in CSF WCC by Day 11 likely reflects effective early in-hospital management (anti-trypanosomal therapy and supportive care) and the natural decline in CNS leukocytosis once parasites are suppressed. In contrast, the directions and magnitudes of some physiologic covariates, for example, respiratory rate should be interpreted cautiously. This is because collinearity with acute illness severity, timing of measurements, or differential missingness can bias single-cohort estimates. These signals are best viewed as hypothesis-generating until prospectively validated.

Recent World Health Organization guidance has expanded oral fexinidazole as a first-line treatment option for *rHAT* and has reduced reliance on routine lumbar puncture for treatment selection. This change increases the relevance of simple admission measurements. Haemoglobin and albumin should therefore be regarded as candidate correlates for prospective evaluation rather than substitutes for clinical assessment or indicated cerebrospinal-fluid investigation.^11,12,22,26,27,28^

Our findings align with the biological and clinical understanding of *rHAT*. Typically, *rHAT* follows an acute, fulminant course with early systemic inflammation and rapid CNS involvement compared with gambiense disease, features consistently described in contemporary reviews and guidelines.^2,11,12,28^ While extensive clinical descriptions of *rHAT* emphasise early neurological and systemic features, no studies quantified routine blood markers alongside CSF indices at presentation.^26,29^ Our findings add empirical support to the concept that simple haematologic and biochemical measures are informative severity surrogates in trypanosomiasis, mirroring their prognostic utility across other severe infections.

This study has limitations. Firstly, it was a small, single-centre retrospective cohort, and the findings may not generalise beyond comparable rural *rHAT* settings. Secondly, of the 187 screened records 144 were excluded because of missing identifiers, absence of cerebrospinal-fluid white-cell counts or missing key laboratory variables; because non-random exclusion may have introduced selection bias. Thirdly, Day-5 cerebrospinal-fluid counts were rarely recorded, limiting assessment of early trajectories. Fourthly, complete-case modelling may have introduced bias if missingness was related to disease severity or clinical management. Fifthly, diagnostic and treatment practices changed over the 2012–2024 study period and were not modelled explicitly. Sixthly, cerebrospinal-fluid white-cell count is an imperfect measure of CNS involvement, and the threshold used in staging is not a biological boundary. Finally, the study assessed association rather than prediction; receiver operating characteristic curves, calibration, action thresholds, positive predictive values and negative predictive values were not evaluated. Prospective multicentre studies using an independent reference standard are required before haemoglobin or albumin can be recommended for clinical triage.

## Conclusion

In patients hospitalised with *rHAT*, lower haemoglobin and albumin were independently associated with higher cerebrospinal-fluid white-cell counts, while counts declined by Day 11 of hospital care. These findings identify two routinely available laboratory measures that reflect CNS inflammatory burden and may complement clinical assessment in district-level settings where lumbar puncture is de-prioritised or delayed. However, this study does not establish diagnostic accuracy or actionable treatment thresholds. Prospective, multicentre validation studies are needed to determine whether haemoglobin and albumin improve triage, referral and monitoring decisions in routine *rHAT* care.
